# Neuron-specific enolase and bispectral index/suppression ratio for prognostication after cardiac arrest

**DOI:** 10.1186/cc12254

**Published:** 2013-03-19

**Authors:** M Van Laer, K Deschilder, P Lormans, J Gillet, W Stockman

**Affiliations:** 1 UZ Leuven, Belgium; 2 HHRM, Roeselare, Belgium

## Introduction

Neuron-specific enolase (NSE) values >33 μg/ml [1] and low bispectral index (BIS) [2] values correlate with bad outcome after cardiac arrest (CA).

## Methods

In this nonblinded prospective study, we observed all CA patients from February 2011 until September 2012 surviving at least 24 hours. NSE was measured between 24 and 72 hours after CA. From October 2011 onward, we recorded BIS and suppression ratio (SR) values as soon as possible after arrival in the ICU. Patients treated with therapeutic hypothermia (TH) (33°C for 24 hours) received cisatracurium. Cerebral Performance Category (CPC) [3] 1 and 2 were considered good outcome, CPC 3 to 5 bad outcome and were recorded after 3 months. Statistical analysis was performed using SPSS statistics 19.

## Results

NSE >45 occurred in 24/68 patients (35.3%) and invariably correlates with bad outcome. The positive predictive value (PPV) NSE >45 for bad outcome is 100%. No patient in this group ever had a GCS ≥12. NSE >33 and <45 occurred in 16/68 patients (23.5%). Thirteen out of 16 patients (81.2%) had bad outcome. However, 7/16 patients (43.8%) woke up at some time (GCS ≥12). NSE <33 occurred in 28/68 patients (41.2%), 17/28 patients (60.7%) had good outcome and 23/28 patients (88.4%) had GCS ≥12 at some time. The PPV NSE <33 for good outcome is 60.7%. The BIS and SR were measured in only 28 patients. Initial BIS ≤10 occurred in 13/28 patients (46.4%) and correlates with bad outcome in 12/13 patients (92.3%). BIS >30 occurred in nine patients, 6/9 (66.7%) had good outcome. Initial SR ≥75 occurred in 11/28 patients (39.3%) and invariably correlates with bad outcome. NSE >25 and SR >60 occurred in 15/28 patients (53.6%) and invariably correlates with bad outcome.

## Conclusion

NSE >45 uniformly correlates with bad outcome after CA. However, we urge caution for the use of intermediate values (33 to 45). In preliminary data, we report that SR >75 might correlate with bad outcome and that combining NSE and SR might improve the predictive value. Also, low NSE and good initial BIS values correlate with preserved cerebral potential and should encourage the clinician.
